# Oxidative Stress in Neurodegenerative Diseases: From Molecular Mechanisms to Clinical Applications

**DOI:** 10.1155/2017/2525967

**Published:** 2017-07-12

**Authors:** Zewen Liu, Tingyang Zhou, Alexander C. Ziegler, Peter Dimitrion, Li Zuo

**Affiliations:** ^1^Radiologic Sciences and Respiratory Therapy Division, School of Health and Rehabilitation Sciences, The Ohio State University College of Medicine, The Ohio State University Wexner Medical Center, Columbus, OH 43210, USA; ^2^Department of Anesthesiology, Affiliated Ezhou Central Hospital, Wuhan University, Ezhou 436000, China; ^3^Interdisciplinary Biophysics Program, The Ohio State University, Columbus, OH 43210, USA; ^4^Department of Biotechnology, Johns Hopkins University, Baltimore, MA 21218, USA

## Abstract

Increasing numbers of individuals, particularly the elderly, suffer from neurodegenerative disorders. These diseases are normally characterized by progressive loss of neuron cells and compromised motor or cognitive function. Previous studies have proposed that the overproduction of reactive oxygen species (ROS) may have complex roles in promoting the disease development. Research has shown that neuron cells are particularly vulnerable to oxidative damage due to their high polyunsaturated fatty acid content in membranes, high oxygen consumption, and weak antioxidant defense. However, the exact molecular pathogenesis of neurodegeneration related to the disturbance of redox balance remains unclear. Novel antioxidants have shown great potential in mediating disease phenotypes and could be an area of interest for further research. In this review, we provide an updated discussion on the roles of ROS in the pathological mechanisms of Alzheimer's disease, Huntington's disease, Parkinson's disease, amyotrophic lateral sclerosis, and spinocerebellar ataxia, as well as a highlight on the antioxidant-based therapies for alleviating disease severity.

## 1. Introduction

Neurodegenerative diseases are characterized by progressive damage in neural cells and neuronal loss, which lead to compromised motor or cognitive function. Common neurodegenerative diseases include Alzheimer's disease (AD), Parkinson's disease (PD), Huntington's disease (HD), amyotrophic lateral sclerosis (ALS), and spinocerebellar ataxia (SCA) [1–4]. These diseases represent a primary health problem especially in the aging population [3, 5]. For example, AD ranks as the sixth leading causes of death in the United States. PD, the second most prevalent neurodegenerative disease, affects 1 to 2% of the population above age of 65 [2, 6–9]. Reactive oxygen species (ROS) are chemically reactive molecules that have been implicated in the pathogenesis of neurodegenerative diseases. They are naturally generated within the biological system, playing important roles in mediating cellular activities such as inflammation, cell survival, and stressor responses as well as many diseases including cardiovascular disorders, muscle dysfunction, allergy, and cancers [10–13]. Due to their reactivity, high concentrations of ROS can lead to cell death or oxidative stress (OS), which is defined as the disruption of balance between pro-oxidant and antioxidant levels [11]. The complex pathogeneses of the neurodegenerative diseases remain largely unknown; however, mounting evidence suggests that ROS may play a critical role as high levels of OS are commonly observed in the brain of patients with neurodegenerative conditions [3, 14].

**Figure 1:**
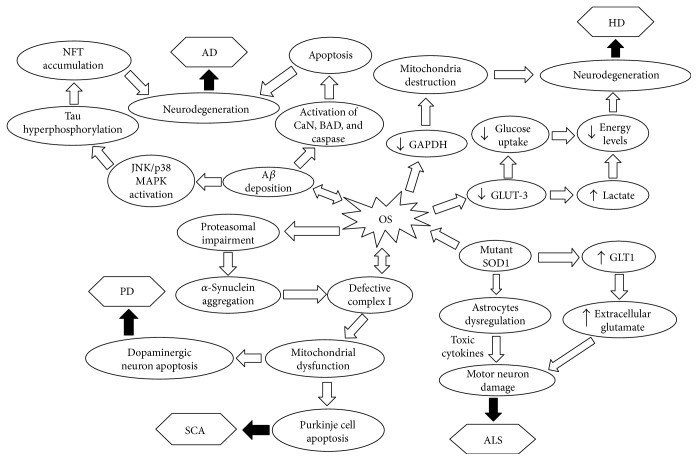
Schematic illustrating the key roles of OS in the development of AD, HD, PD, ALS, and SCA. AD: Alzheimer's disease; ALS: amyotrophic lateral sclerosis; A*β*: amyloid beta; BAD: Bcl-2-associated death promoter; CaN: calcineurin; GAPDH: glyceraldehyde-3-phosphate dehydrogenase; HD: Huntington's disease; JNK: c-Jun N-terminal kinase; MAPK: mitogen-activated protein kinase; NFT: neurofibrillary tangle; OS: oxidative stress; PD: Parkinson's disease; SCA: spinocerebellar ataxia; SOD: superoxide dismutase.

Numerous studies have been performed to investigate the roles of ROS in neurodegenerative progression and some positive results are yielded [15–17]. Although ROS may not be the triggering factor for neurodegenerative diseases, they are likely to exacerbate disease progression through oxidative damage and interaction with mitochondria (Figure 1()) [14]. Of note is that neuron cells are particularly vulnerable to oxidative damage because of their high polyunsaturated fatty acid content in membranes, high oxygen consumption, and weak antioxidant defense [17]. Under physiological conditions, ROS generated from mitochondria, NADPH oxidase (Nox), and xanthine oxidase (XO), are maintained at relatively low levels by endogenous antioxidants [11]. However, the redox balance can be disturbed by neural inflammation or abnormal mitochondrial function [17, 18]. The pathogenesis of several neurodegenerative disorders such as AD and PD is associated with the accumulation of misfolded proteins. The aggregation of these modified proteins can in turn trigger inflammatory response in the brain, which induces marked ROS release and subsequent OS [11, 19]. Mitochondrial dysfunction, which often accompanies aberrant ROS production, is tightly linked with neurodegenerative disorders [3, 20–22]. For example, in HD, mutant huntingtin (mHTT) may directly interact with mitochondria, leading to compromised energy supply and increased ROS levels [23].

Considering the pivotal roles of OS in neurodegenerative diseases, the manipulation of ROS levels may represent a promising treatment option to slow down neurodegeneration and alleviate associated symptoms. In this regard, many compounds that possess antioxidant properties including glutathione (GSH), vitamin C, vitamin E, and coenzyme Q10 have been examined for their potential to attenuate neurodegenerative symptoms; however, mixed results are yielded. For instance, treatment with vitamin E at a dose of 2000 IU per day for two years has been shown to attenuate the functional decline in patients with moderate AD [24]. Vitamin E supplementation early in life potentially decreases the risk of PD [25]. However, another study by Pappert et al. indicated that oral intake of vitamin E for five months had no effect on the elevation of vitamin E levels in ventricular cerebrospinal fluid in PD patients [26]. This suggests the drawbacks of oral administration of antioxidants associated with limited entry across blood–brain barrier and rapid antioxidant metabolism [26]. To counteract this, some small antioxidant molecules, such as ferrostatin-1 (Fer-1), have been developed, which show efficacy in attenuating neurodegenerative symptoms [27, 28]. Furthermore, the time, the method, and the dose of antioxidant administration as well as the stage of the disease are all key factors that should be taken into account when investigating the effects of antioxidant therapies in future studies [29, 30]. Current understanding of the molecular pathogenesis of neurodegeneration is incomplete. Future studies that explore the implications of ROS in various neurodegenerative diseases may be critical to discover novel and reliable therapies.

Currently, no updated systemic review has been performed that specifically focuses on the critical role of ROS and oxidative damage in major neurodegenerative diseases including AD, PD, HD, ALS, and SCA. Thus, there is an urgent need for such comprehensive review. In this article, we have thoroughly discussed the most recent and novel advance in the field of neurodegenerative diseases. Particularly, we highlighted the molecular mechanisms of ROS and the importance of antioxidant therapy in these important neuronal diseases. These critical integrations of various neurodegenerative disease models may shed light into the discovery of novel therapy in the future.

## 2. Oxidative Stress and Alzheimer's Disease (AD)

AD, the most prevalent neurodegenerative disorder, is characterized by the progressive deterioration of behavior, cognition and functionality, which significantly impairs daily living activities [31]. The pathophysiology of AD is mainly associated with the extracellular deposition of amyloid beta (A*β*) plaques and the accumulation of intracellular tau neurofibrillary tangles (NFT) [32, 33]. A*β* plaques can deplete calcium ions (Ca^2+^) storage in endoplasmic reticulum (ER), resulting in cytosolic Ca^2+^ overload. In response to cytosolic Ca^2+^ increase, endogenous GSH levels are reduced and ROS can be overaccumulated inside the cells [34]. ROS-induced OS is emerging as an important factor in pathogenesis of AD as ROS overproduction is thought to play a critical role in the accumulation and deposition of A*β* in AD (Figure 1()) [35]. Mitochondrial dysfunction can lead to ROS misregulation, reduced production of adenosine triphosphate (ATP), altered Ca^2+^ homeostasis, and excitotoxicity. All these alterations may be implicated in the progression of AD [36]. The severe OS observed in AD patients could be a result of overactivating N-methyl-D-aspartate-type glutamate receptors (NMDARs). NMDAR activation has been shown to lead to excessive influx of Ca^2+^ by promoting cell permeability and subsequent generation of neurotoxic levels of ROS/reactive nitrogen species (RNS) [37, 38]. ROS potentially play a role in mediating JNK/stress-activated protein kinase pathways. The activation of these cascades has been implicated in the hyperphosphorylation of tau proteins and A*β*-induced cell death [39]. In addition, A*β* proteins can directly initiate free radical formation via the activation of NADPH oxidase [40]. A*β*-induced ROS overproduction modifies cellular signaling pathways and initiates tau hyperphosphorylation via the activation of p38 mitogen-activated protein kinase (MAPK). An abnormal accumulation of hyperphosphorylated tau proteins can lead to intracellular NFT formation (Figure 1()) [41, 42]. Furthermore, A*β* has been shown to play an important role in mediating cellular apoptotic cascades [43]. Specifically, A*β* can enhance the activity of calcineurin, which in turn triggers Bcl-2-associated death promoter, leading to cytochrome *c* release from mitochondria [44]. A*β* may also directly associate with caspases, and eventually induce neuron apoptosis (Figure 1()) [44].

Aging, inflammation, environmental stress, and some nutritional factors (e.g., redox-active metals) can induce additional OS leading to an increased A*β* production [45–48]. Elderly individuals are more prone to OS, which partially accounts for AD susceptibility in aging populations [45, 49]. Inflammation is responsible for increased expression of cytokines, ROS levels, and cellular toxicity, thereby exacerbating AD progression [50, 51]. A*β* deposition results in microglial activation [52]. It is becoming increasingly evident that prolonged activation of microglia leads to the release of proinflammatory cytokines, initiating a proinflammatory cascade and subsequently contributing to neuronal damage and loss [53]. OS can be induced by environmental factors such as pollutants, chemicals, and radiation [47, 54]. For instance, ROS formation increases when excess iron deposits are present [54]. A*β* itself can interact with metal ions to generate free radicals, and methionine 35 plays a critical role in these reactions [54, 55]. Interestingly, Cu^2+/^Zn^2+^-bound A*β* has been shown to possess a structure similar to superoxide dismutase (SOD), with potential antioxidant properties [56]. Therefore, the supplementation of Cu^2+^ and Zn^2+^ has been suggested as a novel strategy to decrease A*β*-induced ROS generation and metal-catalyzed A*β* deposition [56].

Therapeutic approaches for AD are focused on decreasing A*β* oligomers and phosphorylated tau levels, reducing OS, and controlling epigenetic changes [57, 58]. AD treatments mostly rely on compounds that have neuroprotective, anti-inflammatory, and antioxidant properties [59]. Medications (e.g., rutin, resveratrol, and vitamin E) that target ROS-mediated cascades such as JNK and NF-*κ*B have produced some positive results both in vitro and in vivo [31]. Important factors such as reaction kinetics and bioavailability (absorption, retention in the targeted area, distribution, and transport) must be considered when using antioxidants [59]. Several ROS-related neuroprotective therapeutic strategies continue to show great potential in treating AD. For instance, the nuclear factor erythroid 2-related factor 2 (Nrf2)-mediated antioxidant response element (ARE) pathway is known as an essential protective mechanism against OS [60]. The binding of Nrf2 to ARE activates the expression of several antioxidant genes in a synchronized manner that can work together for oxidative detoxification. Weakened Nrf2-ARE pathways were observed in the brains of transgenic mice with AD symptoms, while the enhancement of Nrf2-ARE cascades using adenoviral Nrf2 gene transfer has shown protective effects against the toxicity of A*β* deposition [60]. Therefore, the transcriptional manipulation of the endogenous antioxidants may hold great promises in relieving AD symptoms [60]. In addition, traditional Chinese medicine and Ayurvedic treatments, metal ion chelators, and histone deacetylase inhibitors have also demonstrated potentials in AD treatment [61–64].

## 3. Huntington's Disease (HD) and Reactive Oxygen Species

HD is a progressive neurodegenerative disease linked with unstable expansion of cytosine, adenine, guanine (CAG) repeats in the HTT gene [65, 66]. The expansion of CAG repeats within the exon1 of the HTT gene gives rise to a mutation that leads to the elongation of polyglutamine tract, resulting in a HTT protein product that is susceptible to aggregation [65]. The mHTT aggregates are accumulated throughout the brain of the affected individuals, which can interrupt protein quality control and transcription process. Those alterations are potentially responsible for the aberrant motor and cognitive problems in HD [65]. Existing HD treatments help to suppress the severity of symptoms; however, no treatment directly cures or significantly halts the disease progression [65, 66]. mHTT is a well-studied mutant of HD that contributes toward the buildups of cytoplasmic plaque and neuronal nuclear inclusion in HD [67–69]. mHTT has been shown to reduce the expression of peroxisome proliferator-activated receptor-*γ* coactivator-1*α*, altering the level of antioxidant enzymes and lowering the concentration of striatal mitochondria [66, 70, 71]. Interestingly, despite the established connection of the role of OS in HD, trials attempting to treat the disease using classic antioxidants have largely been ineffective [72].

The effect of HD on energy levels in the brain has been an area of focus for researchers. Initial studies support the theory that HD lowers energy levels, as reduced glucose usage and increased lactate levels are observed in HD patients [67, 73]. Recent research shows that oxidative damage is related to decreased expression of glucose transporter (GLUT)-3, resulting in inhibited glucose uptake and accumulation of lactate (Figure 1()) [73, 74]. The majority of ATP synthesis occurs through the generation of the proton motive force during operation of the electron transport chain [75]. mHtt has been shown to play a key role in mitochondrial dysfunction. Under electron microscopy, Panov et al. identified that the N-terminal of mHtt can interact with mitochondrial membranes, causing mitochondrial calcium abnormalities. In addition, mHtt has direct inhibitory effects on respiratory complex II [76, 77]. The inhibition of the mitochondrial electron transport can result in higher levels of ROS, as well as decreased ATP production [20, 77]. In 2015, a new mechanism underlying mitochondrial damage in HD was proposed, which suggested that OS could inactivate the catalytic activity of glyceraldehyde-3-phosphate dehydrogenase (GAPDH). The inactive GAPDH (iGAPDH) associates with damaged mitochondria and acts as a signaling molecule in order to drive the impaired mitochondria towards selective degradation via lysosome engulfment. Nevertheless, in the presence of mHtt, iGAPDH can abnormally interact with the long polyglutamine of mHTT at the mitochondrial outer membrane, which blocks the iGAPDH signaling for degradation. Consequently, damaged mitochondria cannot be engulfed by lysosome and unfavorably accumulate in the mHtt-expressed cells, leading to cell death (Figure 1()) [78]. Both mitochondrial alterations and ROS can promote positive feedback loops, resulting in more OS and neuronal loss in the striatum and cortex [66]. Although the involvement of mitochondrial alterations and ROS overproduction in HD progression is well evident, it is not clear which event occurs initially [66, 79, 80].

Numerous studies illustrate the connection between increased levels of oxidative markers and irreversible neuronal damage [65, 81, 82]. One study aimed to establish the merits of neurorehabilitation exercise by tracking the concentrations of 8-hydroxy-2-deoxyguanosine (8-OHdG) and neuron-specific enolase (NSE), both of which are known biomarkers of oxidative damage in HD [83]. In addition, Cu/Zn-SOD (SOD1) was indicated as a potential peripheral biomarker of oxidative damage in neurons and the level was significantly increased in HD patients compared to control, representing a compensatory response to increased oxidative levels in HD [83]. However, whether SOD1 can be considered as an oxidative biomarker in HD remains undetermined because mixed results have been yielded regarding the concentrations and activities of SOD in HD [72, 84]. Upon completion of the neurorehabilitation program (an exercise regimen lasts for three weeks), markedly decreased levels of NSE and 8-OHdG were observed while levels of SOD1 remained high, suggesting the possible neuroprotective role of SOD1 in scavenging free radicals [83]. Overall, physical exercise is recommended for individuals with HD as it can potentially improve redox homeostasis and help to combat disease progression [83, 85, 86].

Other commonly used oxidative biomarkers in HD models include protein carbonyls, thiobarbituric acid reactive substances (TBARS), and 3-nitrotyrosine [72, 87, 88]. In addition, increased F_2_-isoprostane (F_2_-IsoP) levels have been observed in the brain tissue and cerebrospinal fluid of several neurodegenerative disorders including HD and AD. Therefore, the measurement of F_2_-IsoPs provides a potential approach to evaluate the significance of OS in HD patients. Of note, in the early stage of HD progression, there might be an overlap of F_2_-IsoP levels between the HD and control groups [89]. Therefore, the alterations of oxidative biomarkers should be interpreted with caution because OS can also be correlated with other particular factors such as aging or pathological conditions. Moreover, levels of oxidative modifications may not provide sufficient information on whether the oxidative modifications play a causal role in disease progression or directly resulted from cell death [11, 72, 89, 90]. Despite the presence of such limitations, progress has been made in understanding the relationship between OS and HD [72]. The use of robust biomarkers will be crucial to help clarify the contribution of OS in neurodegenerative diseases and to improve the treatment therapies for HD.

## 4. Oxidative Stress and Parkinson's Disease (PD)

PD is the second most prevalent neurodegenerative disease characterized by dopaminergic neuron loss in the substantia nigra pars compacta of the brain [7, 91, 92]. About 1-2% of the population over 65 is affected by PD and this rate increases to 4% in individuals above 85 years of age [6–8]. The pathological mechanisms underlying the degeneration of dopaminergic neurons have been correlated to overaccumulation of ROS or other free radicals. As mentioned previously, excessive ROS production can be caused by mitochondrial dysfunction or inflammation [14]. The maintenance of redox homeostasis is critical for the proper function of redox-sensitive signaling proteins in neuron cells as well as neuronal survival [93, 94]. In the brain, the primary sites of ROS generation include mitochondria in the neurons and glia [14]. The production of these free radicals is exacerbated in PD due to neuroinflammation, dopamine degradation, mitochondrial dysfunction, aging, GSH depletion, and high levels of iron or Ca^2+^ [14, 95]. Furthermore, ROS accumulation in PD may be worsened when the individuals are exposed to environmental factors such as pesticides, neurotoxins, and dopamine [96]. This is evidenced by the significant association between increased risk of PD occurrence and pesticides exposure [96]. ROS have been shown to contribute significantly to dopaminergic neuronal loss (Figure 1) [14, 95]. Other studies suggested that the loss of dopaminergic neurons could also be associated with the presence of neuromelanin since highly pigmented neurons are more susceptible to damages [97]. The formation of neuromelanin appears to be related to dopamine auto-oxidation, a process induced by ROS overproduction [97].

Neurodegeneration-induced ROS can damage key cellular proteins and disrupt lipid membranes, promoting OS. Mitochondrial dysfunction leads to increased free radical production in the respiratory chain [14]. Particularly, mitochondrial complex I deficiency has been identified to strongly associate with PD. Indeed, a large amount of the unfavorable neural apoptosis observed in PD can be attributed to the complex I defect [98, 99]. This defect is correlated with the mutation of PTEN-induced putative kinase 1 (PINK1). PINK1 protein is universally expressed in human tissues and plays a critical role in fighting against OS and maintaining mitochondrial membrane potential [98, 100]. The mutation of PINK1 is related to PD onset [98]. In addition to PINK1, other mutations have been shown to implicate in the development of PD, such as DJ-1, parkin, *α*‐synuclein, and leucine-rich repeat kinase 2 (LRRK2). These mutations can potentially impair mitochondrial function, leading to aggravation of ROS generation and increased susceptibility to OS. For instance, mutated parkin may contribute to the pathogenesis of autosomal recessive PD due to its critical roles in suppressing ROS and preventing the formation of neurotoxic proteins caused by ubiquitination [98, 101]. Furthermore, the aggregation of *α*‐synuclein has been shown to interrupt mitochondrial complex I activities, thus resulting in compromised ATP synthesis and mitochondrial dysfunction [102]. Several mechanisms may account for the accumulation of *α*‐synuclein in PD including decreased efficiency of protein degradation caused by proteasomal impairment as well as translation and posttranslation-associated protein overexpression [102]. Proteasomal impairment, which is partially caused by dopamine-derived ROS, has been shown to play a critical role in the neurodegeneration in PD (Figure 1) [102].

Even though there are currently no effective means for curing PD, the understanding of ROS-related mechanisms in disease progression provides important insight to possible treatments that alleviate PD symptoms. Various neuroprotective strategies have been identified to diminish mitochondrial OS within dopaminergic neurons. Fruits and antioxidants are known to attenuate the damaging effects of free radicals [11, 103]. Vitamins C, E, and GSH are essential antioxidants that can be recycled by antioxidant lipoic acid (LA). LA protects neurons against OS and OS-induced mitochondrial dysfunction by GSH generation and stimulation of lipid peroxide depletion [98, 104]. It has been shown that treatment with LA leads to neuroprotection in an animal study, by improving ATP efficiency and motor coordination [105]. Specifically, rotenone-parkinsonian rats administrated with LA showed reduced lipid peroxide in the brain and exhibited improved motor performance [105]. Substances with antioxidant properties such as coenzyme Q10, vitamin C, tocopherol (vitamin E), docosahexaenoic acid (DHA), *Ginko biloba*, and polyphenols have been tested but have not demonstrated legitimate evidence of any efficacy in neuroprotectivity [14, 106–109]. Failures from those antioxidant treatments should provide future guidance for using combination therapies targeting mitochondrial function enhancement and blocking ROS generation to PD patients [110].

## 5. Molecular Mechanisms of ROS in Amyotrophic Lateral Sclerosis

Amyotrophic lateral sclerosis (ALS) is characterized by progressive loss of motor neurons in the anterior horn of the spinal cord [111, 112]. It is classified as either familial or sporadic depending on whether there is a clearly defined, inherited genetic element. Sporadic ALS (sALS) typically emerges between 50 and 60 years old [113]. The onset of sALS is unknown, and thus the identification of causal genes and environmental factors remains elusive. In familial ALS, about 20% of the cases resulted from mutations in SOD1 [114]. The functions of SOD1 are diverse and include scavenging excessive superoxide radical (O_2_^•–^), modulating cellular respiration, energy metabolism, and posttranslational modification [115]. Although SOD dysfunction leads to a loss of antioxidant capability, evidence has shown that the genetic ablation of SOD1 in mice does not lead to neurodegenerative conditions [11, 16]. In contrast, the gain-of-function of mutant SOD1 protein has been significantly implicated in the motor neuron diseases [16]. For instance, a recent study by Bastow et al. showed that mutant SOD1 can disturb the amino acid biosynthesis of cells in a yeast model and mediate cellular destruction, accounting for the neural degeneration in ALS (Figure 1()) [116].

SOD1 regulates Rac1 directly via the association within endosomes, which subsequently activates Nox. Nox-containing endosomes, also called redoxosomes, are important in regulating proinflammatory signals through NF-*κ*B. Nox forms O_2_^•–^, from molecular oxygen, which plays important roles in cell signaling, enzyme regulation, and antibacterial effect [11, 117]. Under physiological conditions, the ratio of ROS to anti-oxidative molecules is balanced. However, during disease states, rapid changes in ROS levels and disruptions in antioxidant capability can result in increased DNA damage, lipid peroxidation, and apoptosis [11]. SOD1 is responsible for the conversion of O_2_^•–^ into hydrogen peroxide (H_2_O_2_) and molecular oxygen. SOD1 mutants enhance the production of Nox2-dependent ROS, which is thought to be the cause of motor neuron death in ALS [117]. Interestingly, oxidized or misfolded wild-type SOD1 has been shown to lead to mitochondrial dysfunction contributing to the pathogenesis of sALS [118].

Mutant SOD1 can contribute to the progression of familial ALS through the dysregulation of signal transduction pathways in motor neurons and in the activity of supportive glial cells [117, 119]. For example, SOD1 is thought to be an important cell-signaling molecule with neuromodulatory properties. Studies in vitro and in transgenic mouse models show that SOD1 is secreted via the microvesicular secretory pathway. Secreted SOD1 binds to muscanaric receptors on neighboring neurons and increases ERK/AKT signaling, and intracellular Ca^2+^ concentration [120–122]. Whether the secretion of SOD1 is induced by ROS or is constitutive remains as a topic of debate [120]. SOD1 maintains the integrity of motor neurons through activating ERK/AKT signaling, and it has been shown that SOD1 secretion can be increased in neurons under OS [123, 124]. In rats, propofol conditioning treatment was shown to prevent ischemia–reperfusion damage of the spinal cord by increasing PI3K/AKT signaling mediated potentially through increased SOD1 activity [125]. Additionally, OS can induce neuron cell death by inhibiting neuroprotective IGF-I/AKT pathway, prompting further investigation on the role of AKT signaling in neurodegeneration [126].

Like many of the other ALS genes, SOD1 is also expressed in cells other than motor neurons [119]. SOD1 mutant potentially alters astrocytes and microglia activities, contributing to motor neuron degeneration. This pathological mechanism has been termed the non-cell autonomous pathway [119]. The importance of the interaction between astrocytes and motor neurons can be observed using coculture models, where primary or embryonic stem cell derived motor neurons are cultured in the presence of astrocytes expressing mutant SOD1. These cocultured motor neurons, as well as motor neurons treated with conditioned cell culture medium from astrocytes expressing mutant SOD1, experienced increased cell damage [127]. Studies also indicate the excretion of specific soluble factors that are toxic to motor neurons and lead to an inflammatory response, as a direct cause of disease progression [127, 128]. Furthermore, astrocytes expressing mutant SOD1 reduce the expression of glutamate transporter (GLT) 1, leading to increased extracellular concentration of glutamate and glutamate toxicity in motor neurons (Figure 1()) [129, 130]. However, the overexpression of GLT1 via adenoviral vectors is not effective in reducing motor neuron death, supporting the multifaceted role of SOD1 in disease progression of ALS [128].

## 6. Oxidative Stress in Spinocerebellar Ataxia Disease

Spinocerebellar ataxia (SCA) is an autosomal dominant disease characterized by progressive neurodegeneration. Common symptoms associated with SCA are ataxic gait, osculomotor disorders, dysarthria and cognitive impairment, which can ultimately cause death. Over 20 types of SCAs have been identified based on genetic descriptions [131–133]. An expansion of repeated CAG trinucleotides accounts for the major pathogenic mutation in SCA [132]. This mutation leads to the overexpression of mutant ataxin 1 (ATXN1) protein that has an expanded polyglutamine. Mutant ATXN1 can affect the stability of RAR-related orphan receptor alpha (ROR*α*), which plays an important role in Purkinje cell functions. Decreased gene expression of *RORα* has been associated with ataxia and cerebellar hypoplasia [134, 135].

While most types of SCAs are suggested as genetic diseases correlated with ATXN mutation, other pathogenic mechanisms that involve the dysfunction of mitochondria have been proposed [134, 135]. Hakonen et al. observed respiratory complex I deficiency and mitochondrial DNA depletion in the brain of infantile-onset SCA individuals [136]. Although low levels of ROS play a critical role in cell signaling, the overaccumulation of ROS can be neurotoxic and have been implicated in neurodegeneration [11]. A study conducted by Stucki et al. found elevated OS and significant mitochondrial alterations in the Purkinje cells of SCA1. They suspect that there is a potential correlation between the progression of SCA and mitochondrial impairments caused by OS (Figure 1()) [135]. Correspondingly, the study examined the effects of MitoQ (a mitochondrial antioxidant) using a SCA mouse model. It was found that long-term administration of MitoQ significantly restores mitochondrial morphology and functions in Purkinje cells as well as alleviates SCA1-related symptoms such as motor incoordination [135]. Their findings show the potential of using mitochondria-targeted antioxidants as a treatment for SCA1.

Like many neural disorders mentioned previously, SCA is linked with mitochondrial dysfunction [21, 137]. A typical example is the Friedreich ataxia, which is characterized by a loss of frataxin, an iron transporter protein located on mitochondrial inner membrane. With the reduction of the frataxin, iron concentration increases in the mitochondrial matrix, which promotes the conversion of H_2_O_2_ to **˙**OH through the Fenton reaction. The highly reactive **˙**OH molecules can cause oxidative damage to mitochondria and compromise the efficiency of energy production in neuron cells [21, 22]. As a result, antioxidant treatment including vitamin E and coenzyme Q10 supplementation has been shown to improve energy generation for some Friedreich ataxia patients by restoring mitochondrial function and attenuating OS [138].

Due to the high mitochondrial content in the brain, mitochondrial dysfunction can result in significant negative effects on the neural system. ROS are naturally produced from the mitochondrial respiratory chain and play an important role in maintaining mitochondrial function as well as the robustness of neural cells. However, scant research has been done so far to resolve the potential roles of ROS on SCA diseases and develop optimal therapeutic strategies. Further studies are needed to understand the redox mechanisms underlying different types of SCAs, with a focus on ROS-targeted therapies.

## 7. Summary and Prospective

Numerous studies have been performed to investigate the therapeutic effects of antioxidants on neurodegenerative disorders but they have yielded mixed results [14, 106, 110]. This is potentially due to inadequate dosage or timing of administration, or the unsuitable duration of therapy [30, 139]. Novel antioxidant compounds have been developed that show great potential in mediating disease phenotypes. For example, Fer-1, as a potent antioxidant, can effectively prevent neuronal cell death in HD and PD, via the inhibition of lipid peroxidation and the attenuation of glutamate toxicity [27, 28]. Antioxidants protect the redox balance of neural cells by targeting both the upstream and downstream of OS [140]. The upstream protective effects rely on the prevention of ROS overaccumulation and lipid or protein oxidation [11]. Common dietary antioxidants such as vitamins C and E can interact with oxyradicals directly to diminish their toxic effects [140]. Alternatively, supplemental fatty acids can replace oxidized membrane lipids thus reducing oxidative damage [140]. By contrast, antioxidant therapeutics that work downstream of OS focuses on reducing the toxicity from mutant protein aggregation and alleviating neural inflammation [140]. For example, *Ginkgo biloba*, a Chinese herb with strong antioxidant properties, has been shown to be able to improve cognitive conditions in AD by alleviating toxicity of A*β* plaques [141].

Presumably, there is more than one type of ROS involved in the pathophysiology of disease progression [30, 72]. ROS production is subtly regulated by complex antioxidant defense systems within the biological system [11]. Therefore, single exogenous antioxidant supplementation may not be sufficient to combat OS induced under pathophysiological conditions and may even result in a disturbance of redox balance in the body. In this regard, a combination of various antioxidants should be considered for treating neurodegenerative diseases in the future studies. Furthermore, the optimized time, dose and duration of antioxidant treatments remain to be determined for optimal clinical outcomes [30, 72].

In this review, we summarized the complex roles of ROS in common neurodegenerative disorders including AD, PD, HD, ALS, and SCA. High levels of OS have been implicated in many neurodegenerative conditions. Accumulating evidence suggests that ROS may be generated via various mechanisms and have complex roles in promoting disease development. Particularly, mitochondria dysfunction is linked with sustained OS in neurodegenerative disorders. Multiple studies have been performed to explore the effectiveness of antioxidants in attenuating neurodegenerative symptoms. Although there has been no convincing evidence showing their neuroprotective efficacy, research has certainly achieved encouraging outcomes. Further research is paramount in addressing the exact roles of ROS in various neurodegenerative conditions and developing antioxidant-based therapeutic interventions.
